# Immunometabolic Phenotype Alterations Associated with the Induction of Disease Tolerance and Persistent Asymptomatic Infection of *Salmonella* in the Chicken Intestine

**DOI:** 10.3389/fimmu.2017.00372

**Published:** 2017-04-04

**Authors:** Michael H. Kogut, Ryan J. Arsenault

**Affiliations:** ^1^USDA-ARS, SPARC, College Station, TX, USA; ^2^Department of Animal and Food Sciences, University of Delaware, Newark, DE, USA

**Keywords:** *Salmonella enterica*, chickens, disease resistance, disease tolerance, immunometabolism

## Abstract

The adaptation of *Salmonella enterica* to the eukaryotic host is a key process that enables the bacterium to survive in a hostile environment. *Salmonella* have evolved an intimate relationship with its host that extends to their cellular and molecular levels. Colonization, invasion, and replication of the bacteria in an appropriate host suggest that modification of host functions is central to pathogenesis. Intuitively, this subversion of the cell must be a complex process, since hosts are not inherently programmed to provide an environment conducive to pathogens. Hosts have evolved countermeasures to pathogen invasion, establishment, and replication through two types of defenses: resistance and tolerance. Resistance functions to control pathogen invasion and reduce or eliminate the invading pathogen. Research has primarily concentrated on resistance mechanisms that are mediated by the immune system. On the other hand, tolerance is mediated by different mechanisms that limit the *damage* caused by a pathogen’s growth without affecting or reducing pathogen numbers or loads. The mechanisms of tolerance appear to be separated into those that protect host tissues from the virulence factors of a pathogen and those that limit or reduce the damage caused by the host immune and inflammatory responses to the pathogen. Some pathogens, such as *Salmonella*, have evolved the capacity to survive the initial robust immune response and persist. The persistent phase of a *Salmonella* infection in the avian host usually involves a complex balance of protective immunity and immunopathology. *Salmonella* is able to stay in the avian ceca for months without triggering clinical signs. Chronic colonization of the intestinal tract is an important aspect of persistent *Salmonella* infection because it results in a silent propagation of bacteria in poultry stocks due to the impossibility to isolate contaminated animals. Data from our lab promote the hypothesis that *Salmonella* have evolved a unique survival strategy in poultry that minimizes host defenses (disease resistance) during the initial infection and then exploits and/or induces a dramatic immunometabolic reprogramming in the cecum that alters the host defense to disease tolerance. Unfortunately, this disease tolerance results in the ongoing human food safety dilemma.

## Introduction

### *Salmonella* Infection and Poultry

Foodborne illness is a significant worldwide public health problem that continues to plague the world, costing approximately $152 billion annually (1). Despite control efforts that cost over a half a billion dollars annually, foodborne illnesses due to *Salmonella* and *Campylobacter* increased during the last 15 years. In 2013, 20% of the 9.4 million episodes of foodborne illnesses were attributed to *Salmonella* and accounted for 26% of the hospitalizations (2). In 2012, the Foodborne Diseases Active Surveillance Network found that *Salmonella* accounted for over 28% of the confirmed foodborne disease cases in the U.S., and cost U.S. residents $14.6 billion annually, respectively (3). Clearly, efforts to elucidate and implement new and existing methods for control are well justified by the economic cost alone to control *Salmonella, Campylobacter*, and other foodborne pathogens. Poultry products have been associated frequently and consistently with the transmission of enteric pathogens, including *Salmonella* and *Campylobacter* (4).

Salmonellosis is a zoonotic disease caused by the Gram-negative facultative anaerobic, enteric bacterium *Salmonella*. With more than 2,500 serotypes having been described, most *Salmonella* serovars are not restricted to particular host species and are able to colonize the alimentary tract of animals without production of disease (5). Not coincidentally, the most common human clinical isolates, *Salmonella enterica* serotypes Typhimurium (STm) and Enteritidis (SE), are the most commonly detected serotypes in poultry (6).

In poultry, *S. enterica* serotypes can be divided into two groups based on their host species range and their disease pathogenesis (5, 7, 8) with *S. enterica* serotypes *S*. Gallinarum and *S*. Pullorum being chicken-specific and the broad host serovars best exemplified by STm and SE. STm and SE are major causes of zoonotic gastroenteritis in a wide range of host species worldwide (5–8).

Both broad host range *Salmonella* serovars (STm and SE) are able to colonize the gastrointestinal tract of chickens a few days of age without clinical disease but induce a rapid (within 4 h) and mild acute inflammatory response (9). After oral infection of fowl, the bacterial colonization is durable in the gut where the two ceca represent a suitable site for colonization. *Salmonella* can be transmitted horizontally within the flock after fecal shedding as well as vertically through the trans-ovarian route. Chicks are more susceptible to salmonellosis than adults. In particular, asymptomatic carriers have a major role in *Salmonella* propagation in poultry and hence in food contamination, since they cannot be easily isolated and identified. The persistence of *Salmonella* in the intestinal tract of chickens is the main cause of disease propagation in poultry (5, 8, 9).

## Persistence of *Salmonella* Intestinal Colonization

Infection with a pathogenic microorganism usually results in the host responding by activating the innate and adaptive immune responses. However, some pathogens, such as *Salmonella*, have evolved the capacity to survive the initial robust immune response and persist (10–12). The persistent phase of infection usually involves a complex balance of protective immunity and immunopathology. The interactions between the host and pathogen are very complex and likely reflect the coevolution and fine tuning of bacterial virulence mechanisms and host immune responses (13). Until recently, very little is known about the molecular regulatory interactions between the host immune response and virulence mechanisms that lead to *S. enterica* persistence in the avian intestine. The carrier state, corresponding to a persistent colonization of the gut, is established, and *Salmonella* is able to stay in the ceca for months without triggering clinical signs (14). Chronic colonization of the intestinal tract is an important aspect of persistent *Salmonella* infection because it results in a silent propagation of bacteria in poultry stocks due to the impossibility to isolate contaminated animals (15, 16).

The establishment of persistence is in the face of a substantial immune response requiring evasion or modulation of the response by the bacteria. The fact that many *Salmonella* serovars persist within the chicken intestinal tract with little sign of gastrointestinal disease despite eliciting a considerable inflammatory response and that inflammatory responses to *Salmonella* are relatively short-lived (17), strongly suggests there is a degree of regulation of this response.

## Host Defense Strategies

Historically, host defense strategy has been based on the outcome of the immune response’s ability to detect and eliminate pathogens through multiple killing mechanisms known as host resistance (18, 19). However, a relatively new immunological concept, tolerance as a host defense strategy has been put forward (19, 20). Tolerance is the ability of the host to limit the damage caused by both the pathogen and the host immune response, i.e., immunopathology (20). Tolerance, as a host defense strategy, has been ignored in veterinary infectious disease studies (18). It is important to point out that infection tolerance is not immune tolerance which is defined as “unresponsiveness of the immune system to substances or tissue that has the capacity to elicit an immune response” (21).

Unlike immune responses that have measureable outputs to evaluate effectiveness, disease tolerance lacks clear-cut outputs (18). However, measurement of local cell metabolic processes and function, redox status, concentrations of metabolites, and organelle function of parenchymal cells and tissues (host’s cells/tissues that do not have a direct impact on pathogens) would be beneficial in evaluating stress and damage responses. Since a pathogen and the induced immunopathology can theoretically affect any physiological system, disease tolerance would involve a number of processes that will reduce host susceptibility to damage. Therefore, any physiological mechanism that typically maintains homeostasis and functional integrity of host tissues could contribute to disease tolerance. Mechanistically, limiting tissue damage is regulated by a number of evolutionarily conserved stress and/or damage responses. These responses confer tissue damage control, by providing cellular adaptation to environmental changes (22). For example, stress responses maintain cellular functions by activating metabolic processes in response to local alterations in oxygen tension (hypoxia), redox status (oxidative stress), osmolarity, and metabolite concentrations (ADP/ATP, glucose). All are essential mechanisms of cell and tissue homeostasis (23). Damage responses attempt to preserve cellular functions while minimizing damage to macromolecules (DNA, lipids, and proteins) and/or organelles (mitochondria, Golgi, and endoplasmic reticulum) (19, 23). The concept of tolerance as a host defense mechanism has led to an excellent recent editorial (24). The authors ask a very provocative question of what effect does therapeutics based on reducing the symptoms induced by a pathogen (tolerance) instead of reducing pathogen numbers have on evolution of the host population and the pathogen? By not reducing pathogen numbers, will there be an effect on pathogen transmission and spread and the potential development of disease carriers or will the limitation of disease symptoms allow the host immune system to concentrate on controlling pathogen numbers?

## *Salmonella*-Chicken Infection Biology

*Salmonella* can be carried by poultry with virtually no ill effects on the host; whereas, in humans, the same bacteria cause pathological inflammation (25, 26). The induction of this severe inflammation appears to be essential for the salmonellae organisms to procure critical nutrients and respiratory substrates from the host allowing the pathogen to out-compete the commensal microbiota that rely on anaerobic fermentation (27–29). Thus, the interactions between the host response and *Salmonella* infections in the intestinal tract of poultry appear to be directed toward disease tolerance characterized by the asymptomatic nature of infection. Therefore, the chicken and bacteria appear to have evolved a relationship that minimizes both the normal host response and the normal bacterial virulence. However, this tolerant state is “detrimental to food safety” in humans (30).

In a recent review, Wigley (9) described how *Salmonella* infection in chickens facilitated our understanding of avian immunology over the last 20+ years. At the end of his review, Wigley (9) asked a “few key questions that still needed to be fully answered.” We have used two of the questions for the basis of our studies into the persistence of colonization of *Salmonella* in the intestine of chickens. Namely, (1) what mechanisms trigger the persistence of *Salmonella* in the cecum and (2) how is the intestinal response regulated to prevent excessive damage to the host? The *Salmonella*-chicken dynamics provide a unique system where the pathogen appears to evade the immune system, alters the local intestinal phylogeny of recognition and signaling pathways, and takes residence amongst the cecal microbiota.

Based on the findings by us and others, we propose that *Salmonella* infection in the chicken can be separated into three distinct stages of host defense strategies characterized by the cecal immune effector cells, immune gene expression, and immunometabolic responses at different times postinfection:

1. Stage 1, Disease Resistance: characterized by an acute heterophil-mediated pro-inflammatory response and anabolic metabolism 1–2 days postinfection.
2. Stage 2, Disease Tolerance: exemplified by a profound an increase in cecal T regulatory cells and an anti-inflammatory response and a conversion to a catabolic phenotype 4 days postinfection.
3. Stage 3, Homeostasis: a return to a homeostatic metabolic phenotype with a more IL-10-mediated regulatory immune response 5–10 days postinfection.

## Stage 1, Disease Resistance

*Salmonella* invasion of the chicken intestine induces an inflammatory process resulting in the expression of pro-inflammatory cytokines and chemokines by epithelial cells lining the intestine (17, 31–33). The outcome of this activation of innate immunity is a major influx of heterophils (granulocytes) to the intestine that limits bacterial invasion (34, 35) but does not lead to a pathological inflammation that is seen in humans (17, 36). However, this heterophil response does not have a significant protective response against the salmonellae bacteria that remain in the lumenal side of the cecal epithelium. Interestingly, this inflammatory response is largely resolved by 3–4 days postinfection (35, 37, 38) characterized by the reduction of pro-inflammatory cytokines mRNA transcription in the cecum to non-infected control levels yet *Salmonella* can persist in the intestine and be shed in the feces for several weeks (17).

Accompanying intestinal inflammation are extreme alterations in tissue metabolism, most of which are due to the incoming heterophils and other inflammatory cells and can include the increase in fatty acid, protein synthesis, glycolysis and the production of reactive oxygen intermediates (38, 39). Energy-demanding processes, such as migration, phagocytosis, and the generation of an oxidative burst, that accompany the recruitment of the heterophils to the site of infection, trigger transcriptional and translational changes in tissue phenotype (predominately the metabolic signaling pathway of mTOR phosphorylation) that shifts fundamental changes to the local intestinal tissue to anabolic metabolism (37–39). Further, the presence of the PMNs and subsequent metabolic requirements exhaust the microenvironmental oxygen to quantities nearing anoxia (39). This localized oxygen depletion leads to the stabilization, and thus the activation of the transcription factor, hypoxia-inducible factor-α (HIF1α), and activation of the HIF1α signaling pathway that resolves inflammation, and potentially provides a more tolerant local setting for the bacteria (40, 41). Under these oxygen-deprived conditions, HIF1α activation inhibits mTOR activity resulting in a potent anti-inflammatory microenvironment through the production and stimulation of T regulatory cells would regulate tissue damage (42, 43).

The initial inflammatory response (disease resistance) is sufficient to help control invasion and elicit the development of a protective acquired immune response that can lead to systemic and eventual clearance of gastrointestinal infection.

## Stage 2, Disease Tolerance

### Immunological Phenotype

It has been demonstrated by numerous groups that early cecal pro-inflammatory (disease resistance) signals following initial infection with STm or SE was dramatically downregulated 2–4 days after infection that is linked with the development of an anti-inflammatory, Th2 response (15, 17, 32, 34, 44) to increased expression of IL-10 and TGF-β, which suggests the end of the disease resistance and the start of a disease tolerant state were being initiated.

It would seem likely that regulation of inflammatory immune responses, presumably by regulatory T cells (Tregs), allows *Salmonella* to persist within the gut for a number of weeks without disease to the bird. Such a “tolerogenic” response would have little or no impact on the bird itself but has public health consequences in allowing persistence for several weeks, particularly given broiler chickens are typically slaughtered at around 5 weeks of age. Subsequently, we have found an expansion of the CD4+ CD25+ T cell (Treg) population in the cecum of *Salmonella*-infected chickens (45). Functionally, the cecal Tregs had increased suppressive activity for T effector cells and had a profound increase in IL-10 mRNA transcription. In the murine model of ST infection, the ability of the bacteria to persist or be cleared has been found to be dependent on the presence and function of Tregs (46).

Mechanistically, in a series of experiments using a chicken-specific kinome array, the plasticity of the local cecal immune phenotype where the initial inflammatory response against a *Salmonella* infection is then followed by a striking alteration in the immune microenvironment 2 days later during the establishment of a persistent *Salmonella* infection (35, 37, 38). We used the power of a species-specific kinome array to delineate the mechanisms that alter the host avian inflammatory responses and uncover host signaling events that are manipulated by the bacteria in order to establish a persistent infection. First, we found that the establishment of a persistent *Salmonella* cecal colonization in chickens activates both the canonical (Smad-dependent) and non-canonical (Smad-independent) TGF-β signaling pathways (35). TGF-β functions by controlling immune responses by suppressing non-Treg function and promoting Treg function. These results are suggestive of a change in the cecal mucosal phenotype from pro-inflammatory to tolerance is, in part, mediated by the increased expression of TGF-β that activates both Smad-dependent and -independent TGF-β pathways that increases the differentiation and function of Tregs while decreasing the function of pro-inflammatory immune cells. Second, during the establishment of a persistent *Salmonella* cecal infection, we found the activation of the non-canonical Wnt signaling pathways (35). Non-canonical Wnt signaling controls nuclear localization of nuclear factor of activated T cell (NFAT) transcriptional factor. NFAT regulates the interaction of the innate immune cells with acquired immunity to promote anti-inflammatory programs and is essential for both development and function of Tregs (47, 48). The transformation in the avian host response from resistance to tolerance during the establishment of *Salmonella* persistence was further confirmed by a study showing two select host immune signaling pathways were altered; namely, the T cell receptor and JAK–STAT signaling pathways (38). Both signaling pathways were shown to have alterations in the phosphorylation of multiple peptides that resulted in the inactivation of an active immune response in the local cecal environment. The response was characterized by the dephosphorylation of phospholipase c-γ1 that induced the dephosphorylation (inhibits activation) of NF-κB signaling, thus preventing activation of immune response genes and the phosphorylation of NFAT signaling which activates anti-inflammatory cytokine production as described above. Further, interferon-gamma production that is central in the resolution of *Salmonella* infections in the cecum of avian species (16, 32, 49) was also found to be inhibited in the cecum of SE-infected chickens through the disruption of the JAK–STAT signaling pathway (dephosphorylation of JAK2, JAK3, and STAT4). The JAK–STAT signaling pathway transmits information from extracellular chemical signals to the nucleus resulting in DNA transcription and expression of genes involved in immunity, proliferation, differentiation, and apoptosis (50, 51). Taken together, by 4 days postinfection, the immune phenotype in the cecum of *Salmonella*-infected chickens has undergone a dramatic alteration in host responsiveness where the host does not appear to recognize the bacterium as a pathogen resulting in a persistent cecal colonization.

### Metabolic Phenotype

Concurrently to the alterations in the local immune response during the tolerance phase, profound metabolic phenotype alterations occurred in the cecal tissue of *Salmonella*-infected chickens from the early resistance response (4–48 h postinfection) which is pro-inflammatory, fueled by glycolysis and mTOR-mediated protein synthesis to the later tolerance phase (4 days postinfection) where the local environment has undergone an immune-metabolic reprogramming to an anti-inflammatory state driven by adenosine monophosphate-activated protein kinase (AMPK)-directed oxidative phosphorylation (37). Therefore, metabolism appears to provide a potential measurement that characterizes a state of infection tolerance. Additionally, these results provide further evidence of what Olive and Sassetti (52) describe as a pathogen’s ability to “sense the metabolic environment of the host, adapting to changing nutrient availability.” Further, these phosphorylation alterations at the gut level during the first 3 weeks after infection of day-old broilers with ST appear to lead to key metabolic changes that affected fatty acid and glucose metabolism through the 5′-AMPK and the insulin/mTOR signaling pathway in the *skeletal muscle* were altered (53). Supplemental proof for the effects of fatty acid and glucose metabolism on long-term persistence of *Salmonella* was recently demonstrated using the murine macrophage model (54, 55). ST preferred living in alternatively activated macrophages that require the activation of the transcription factor, peroxisome proliferator-activating receptor δ (PPARδ), which regulates fatty acid metabolism (54). Thus, the bacteria prefer macrophages that employ oxidative metabolism for energy instead of glycolysis due to the factor that disruption of glycolysis is a signal of the activation of the NLRP3 inflammasome and the subsequent initiation of inflammatory cell death, pyroptosis (55).

## Stage 3, Homeostasis

Immunologically, the third stage of an avian *Salmonella* infection occurs shortly after day 4 postinfection with the expression of a disease tolerance state. The number of Tregs in the cecum of the infected birds remains constant suggesting an immune regulation state further evidenced by the increased transcription of IL-10 and TGF-β (37, 38, 44, 45). The underlying question here is whether *Salmonella* is no longer “sensed” by the immune system as foreign invaded and has become a component of the cecal microbiome. Experiments to answer this question are ongoing in our laboratories.

Metabolically, the local microenvironment appears to go through a final reprogramming during this third stage of infection moving from a catabolic state in stage 2 to a more homeostatic status. This was verified in our kinome studies by the fact that we observed no differences in the metabolic signaling pathways in the ceca from the *Salmonella-*infected and non-infected chickens (37, 38, 44).

## Perspective

The data from our lab and others soundly support the hypothesis that *Salmonella* have evolved a unique survival strategy in poultry that minimizes host defenses (disease resistance) during the initial infection and then exploits and/or induces a dramatic immunometabolic reprogramming in the cecum that alters the host defense to disease tolerance (summarized in Table 1). The ability to induce a state of disease tolerance is unique to the poultry-*Salmonella* interactome in that it allows the bacterium to establish a long-term persistent infection in the cecum while allowing the host to control disease pathology. Unfortunately, it also results in the ongoing human food safety dilemma. It should be pointed out that the energy balance reported in Table 1 is not backed by direct evidence in these experiments but is an assumption based on the fact that AMP is elevated when AMPK is activated and ATP is elevated when mTOR is activated.

**Table 1:** **Immunometabolic alterations in the chicken cecum during the establishment of a persistent infection**.

Parameter	Stage 1 Days 1–2 postinfection Disease resistance	Stage 2 Days 3–4 postinfection Disease tolerance	Stage 3 Days 5–28 postinfection Homeostasis
Cytokines	Pro-inflammatory (17, 31–33)	IL-10, TGF-β (17, 32, 44)	IL-17, IL-10
Immune cells	Heterophils (34, 35), M1 macrophages (55)	Th2 T-helper cells (15), regulatory T cells (Tregs) (45), M2 macrophages (54)	Tregs, APCs
Signaling	Toll-like receptor, NOD receptor, HIF1 (40, 41), NF-κB (38)	Smad, Wnt (35), nuclear factor of activated T cell (47, 48), JAK–STAT (38)	Maintenance
Metabolism	Increased fatty acid synthesis, protein synthesis, glycolysis, reactive oxygen intermediates (38, 39)	Oxidative phosphorylation, fatty acid catabolism (53)	Aerobic
Metabolic signaling	mTOR (37–39)	Adenosine monophosphate-activated protein kinase (37)	Energy neutral
Energy balance	AMP:ATP	AMP:ATP	AMP:ATP
Oxygen state	Hypoxia (43)	Normoxia	Normoxia
Metabolic state	Anabolic	Catabolic	Balanced
Immune state	Inflammation	Anti-inflammatory (tolerance)	Non-inflammatory

These studies have used the emerging field of immunometabolism at the tissue level to identify potential mechanisms by which the host can tolerate a *Salmonella* infection. Recently, an immunometabolic mechanism for disease tolerance to a murine STm infection was found to involve the microbiome and the insulin-signaling pathway (56). Taken together, identifying potential molecular mechanisms of disease tolerance as a host defense can not only “provide a perspective into the evolutionary forces that have driven coevolution” (56) of host–pathogen interactions but also provide the discovery of new therapeutic targets to control foodborne pathogens.

## Author Contributions

MK and RA conducted the experiments and made substantial, direct, and intellectual contribution to the work and approved it for publication.

## Conflict of Interest Statement

The authors declare that the research was conducted in the absence of any commercial or financial relationships that could be construed as a potential conflict of interest.
